# Aerosolized adenovirus-vectored vaccine as an alternative vaccine delivery method

**DOI:** 10.1186/1465-9921-12-153

**Published:** 2011-11-21

**Authors:** Chad J Roy, Alida Ault, Satheesh K Sivasubramani, J Patrick Gorres, Chih-Jen Wei, Hanne Andersen, Jason Gall, Mario Roederer, Srinivas S Rao

**Affiliations:** 1 Infectious Disease Aerobiology, Division of Microbiology, Tulane National Primate Research Center, Covington, LA 70447 USA; 2 Laboratory Animal Medicine, Vaccine Research Center, National Institutes of Health. Bethesda, MD 20895 USA; 3 Vector Core Section, Vaccine Research Center, National Institutes of Health, Bethesda, MD 20895 USA; 4 BIOQUAL, Inc., Rockville, MD 20850 USA; 5 GenVec Inc. Gaithersburg, MD 20878 USA; 6 ImmunoTechnology Section, Vaccine Research Center, National Institutes of Health, Bethesda, MD 20895 USA

## Abstract

Conventional parenteral injection of vaccines is limited in its ability to induce locally-produced immune responses in the respiratory tract, and has logistical disadvantages in widespread vaccine administration. Recent studies suggest that intranasal delivery or vaccination in the respiratory tract with recombinant viral vectors can enhance immunogenicity and protection against respiratory diseases such as influenza and tuberculosis, and can offer more broad-based generalized protection by eliciting durable mucosal immune responses. Controlled aerosolization is a method to minimize vaccine particle size and ensure delivery to the lower respiratory tract. Here, we characterize the dynamics of aerosolization and show the effects of vaccine concentration on particle size, vector viability, and the actual delivered dose of an aerosolized adenoviral vector. In addition, we demonstrate that aerosol delivery of a recombinant adenoviral vaccine encoding H1N1 hemagglutinin is immunogenic and protects ferrets against homologous viral challenge. Overall, aerosol delivery offers comparable protection to intramuscular injection, and represents an attractive vaccine delivery method for broad-based immunization campaigns.

## Introduction

Conventional parenteral delivery of flu vaccines is limited in its ability to induce locally-produced immune responses in the respiratory tract as well as its capacity for efficient widespread distribution [1,2]. Recent studies evaluated intranasal delivery of recombinant vector-based influenza vaccines as an alternative route of delivery that may enhance safety, efficacy, and ease of administration [3-6]. Vaccination in the respiratory tract may enhance protection against respiratory diseases such as influenza, tuberculosis, and measles, and may provide more generalized protection by inducing long-lasting mucosal immune responses [7,8]. Studies have also shown that mucosal immunity induced via intranasal delivery provides cross-protection against heterologous strains [9-15], and enhances heterosubtypic immunity for protection against multiple influenza A subtypes [9,10,16,17]. Other logistical advantages of an intranasal vaccine include the reduced risk of infection and contamination due to the non-use of needles and syringes, and avoiding the need for disposal strategies of sharps after mass vaccination campaigns [18-20].

The currently licensed intranasal vaccine FluMist™ is a live-attenuated virus administered using a Becton-Dickenson AccuSpray™ device which generates a high-speed spray of large vaccine particles, with a mass median aerosol diameter (MMAD) > 70 μm. With particle size and speed being key factors determining aerosol deposition in the airway, these high-speed, large particle sprays are often trapped in the external nares and do not navigate to the internal airways which are the primary target of vaccination. Furthermore, droplets deposited in the nose can drip out or roll back toward the pharynx causing unpleasant sensations, diminishing acceptability of the vaccine [7]. In contrast, controlled aerosolization helps to minimize vaccine particle size variability and ensures delivery to the lower respiratory tract and internal target airways [21]. In animals, aerosol vaccination is currently used globally to immunize poultry against Newcastle disease and shows promise of successful immunization in fowls and pigs against a variety of diseases including fowlpox, infectious bronchitis, hog cholera, pseudorabies, erysipelas, gastroenteritis, pasteurellosis, and mycoplasmosis [19]. Notably, aerosol measles vaccination of 4 million Mexican schoolchildren in 1989-90 demonstrated a seroconversion rate of 52-64% (similar to subcutaneous administration) and an overall efficacy of 96%, with excellent public acceptance and fewer side effects than subcutaneous vaccination [22]. However, while aerosol vaccination shows advantages in eliciting protective immune responses as well as cost-efficacy of administration, more studies are needed to further characterize the method and ensure that it is a safe and practical alternative.

Here, we elucidate the dynamics of aerosolization by analyzing particle size, vector viability, and actual delivered dose of an aerosolized adenoviral vector. This vector has been previously used alone or in combination with DNA prime immunizations to protect against lethal influenza challenges in mice and ferrets [23,24]. In addition, we compare the efficacy of aerosol vaccination to intramuscular (IM) injection of this recombinant adenoviral (Ad) vaccine encoding seasonal H1N1 immunogens against homologous challenge in ferrets.

Results indicate that vaccine concentration influences aerosol size, viability, and actual delivered dose, and should be considered in designing an optimal aerosol vaccination regimen. Results from the influenza challenge study indicate that aerosol vaccination elicits humoral immune responses and protects against H1N1 influenza challenge. Furthermore, the use of aerosol as the modality of vaccination has previously shown minimal to no lasting pathology in the lung, and is comparable to IM injection in immunogenicity and protection.

## Materials and methods

### PARI eFlow^® ^nebulizer device

The PARI eFlow^® ^device is a portable, electronic aerosol platform developed primarily to deliver liquid pharmaceutical therapies in a clinical setting [25-27]. The PARI eFlow^® ^aerosol device generates aerosols via a laser drilled membrane that is actuated via a piezoelectric crystal which pumps liquid through the membrane at relatively high velocity. This device was utilized in this study to deliver a biologic-based vaccine to the respiratory tract.

### Particle size characterization

Empty Ad vector solutions were diluted in final formulation buffer (FFB) at seven different log concentrations, ranging from 10^5 ^to 10^11 ^particle units (PU)/mL, in order to establish size distribution characteristics of the aerosolized particles produced by the PARI eFlow^® ^device. Three samples of 1 mL aliquots of each Ad concentration were aerosolized into a small plexiglass chamber using the PARI eFlow^® ^device. An automated particle sizer (Aerodynamic Particle Size Model 3321, TSI Instruments, St. Paul, MN) was used to sample the generated atmosphere within the chamber and produce size and relative distribution estimates.

### Adenoviral aerosol viability

Effects of the aerosol generation using the PARI device upon the relative viability of the Ad vector were unknown prior to this study. In order to determine the effect of aerosolization on the viability of an Ad vector and estimate the actual inhaled dose, empty Ad vector solutions were diluted at seven different log concentrations (as done in the particle size characterization experiment). Three samples of 1 mL aliquots were aerosolized as above, and the resultant aerosol was collected into an All-Glass Impinger (AGI) (Ace Glass, Vineland, NJ) containing 10 mL phosphate-buffered saline (PBS). The liquid capture of aerosol particles within the impinger contained an air sample that represented the atmospheric concentration of the Ad generated by the aerosol device. Immunofluorescence assays were performed in triplicate on collected samples to measure infectious titers post-aerosolization [28]. A non-aerosolized control for each concentration was also included. The fluorescent forming unit (FFU) results from the sampled aerosols were directly compared with the FFU content of the "pre" aerosol starting concentrations (expressed in FFU/l liquid starting concentration) in order to determine viral efficiencies after aerosol generation. The unitless factor (viral efficiency factor; *F_e_*), defined as a ratio of the starting concentration (FFU/l liquid inoculum) to the aerosol concentration (FFU/l aerosol) was used to express the relative viability of the Ad in aerosol (Table 1). While the magnitude of this value does not have a direct biological interpretation, comparison across conditions would reveal differential viral loss.

**Table 1:** Effect of aerosolization on Ad viability.

	PS01		PS02		PS03	
**C_s _(*FFU/ml*)**	**C_a _*(FFU/ml) ***	**F**_**e**_	**C_a _(*FFU/ml*)**	**F**_**e**_	**C_a _(*FFU/ml*)**	**F**_**e**_

1.00E+08	1.76E+02	1.76E-06	*n.d*.	*n.d*.	5.64E+01	5.64E-07
1.00E+09	2.74E+02	2.74E-07	5.04E+02	5.04E-07	9.05E+02	9.05E-07
1.00E+10	4.77E+03	4.77E-07	1.22E+04	1.22E-06	9.55E+03	9.55E-07
1.00E+11	*n.d*.	*n.d*.	6.25E+04	6.25E-07	1.61E+05	1.61E-06
Mean(PS) ± S.E.	**8.37E-07 ± 2.69E-07**		**7.84E-07 ± 1.28E-07**		**1.01E-06 ± 1.09E-07**	

Aerosol viability estimates for Adf.11D LN05224 from each independent experiment (PS01, PS02, PS03) using logarithmic starting concentrations (C_s_) and the corresponding aerosol concentration (C_a_) obtained from a proximity sampler located within a characterization chamber. The viral efficiency factor (F_e_), a unitless ratio of the C_s _and C_a_, is used as an indicator of the dilution and associated loss of biologic activity during the process of aerosolization of the solution containing the adenovirus during characterization. The overall mean F_e _for all samples attempted was 8.8 × 10^-7^.

### Construction of recombinant Ad5 vaccine encoding hemagglutinin (HA)

An Ad5-based first-generation vaccine expressing HA was constructed as described previously [29]. HA immunogen was derived from the A/Brisbane/59/2007 H1N1 isolate, a strain used in 2008-2009 Flumist™ trivalent intranasal vaccine [30]. Briefly, PacI-linearized shuttle vectors containing the influenza immunogen were recombined with the right side of Ad5 genomic DNA carried in a cosmid by use of Cre recombinase (Novagen, Madison, Wis.). The resulting recombinant was ethanol precipitated, dissolved in Tris-EDTA, and transfected into HEK 293 cells. Recombinant Ads were observed based on plaque formation 10 to 14 days after transfection. Viruses were amplified, purified two times through a CsCl gradient, and stored in PBS plus 15% glycerol at -20°C.

### Ferret immunizations

4-6 month old male, Fitch ferrets (Triple F Farms, Sayre, PA), sero-negative for exposure to currently circulating H1N1, H3N2, and B flu strains were housed and cared for at BIOQUAL, Inc. (Rockville, MD). These facilities are accredited by the American Association for the Accreditation of Laboratory Animal Care (AAALAC) International and meet NIH standards as set forth in the Guidelines for Care and Use of Laboratory Animals [31]. Prior to the start of the study, a temperature transponder (Biomedic Data Systems, Inc., Seaford, DE) was implanted into the neck of each ferret. Two groups of 7 ferrets were immunized twice at weeks 0 and 3 with an Ad5 vaccine encoding the HA gene from the A/Brisbane/59/2007 isolate at a dose of 1 × 10^10 ^PU. One group of ferrets received the Ad5 vaccine via IM injections in the upper thigh muscle while another group received the same vaccine via aerosol delivery using a PARI eFlow^® ^nebulizer (Starnberg, Germany). Control ferrets were also immunized with empty Ad vectors via aerosol delivery.

For aerosol administration, ferrets were anesthetized with IM injections of 5-10 mg/kg ketamine and 0.5-1.0 mg/kg xylazine, and placed in a BSL-2 biosafety cabinet. With the PARI eFlow^® ^device set up according to manufacturer's instructions, 1 mL of the vaccine was poured into the medication reservoir, and a modified facemask was applied to the ferret, ensuring a seal. The device was then activated and automatically deactivated after all of the liquid volume containing the vaccine was expended. The facemask was applied for an additional minute to ensure inhalation of any remaining aerosol.

### Hemagglutination inhibition (HI) assay

Sera were treated with receptor-destroying enzyme (RDE) by diluting one part serum with three parts enzyme and incubated overnight in a 37°C water bath. The enzyme was inactivated by a 30 min. incubation at 56°C followed by addition of six parts PBS for a final dilution of 1/10. HI assays were performed in V-bottom 96-well plates using four hemagglutinating units (HAU) of virus and 1% horse erythrocytes as previously described [32]. Ferret sera were tested against homologous H1N1 influenza strain A/Brisbane/59/2007.

### Production of pseudotyped lentiviral vectors and measurement of neutralizing antibodies

Production of pseudotyped lentiviral vectors for H1N1 and neutralization of pseudotyped viruses were performed as previously described [33].

### Ferret challenge experiments

Ferrets were challenged three weeks after their second immunization with ~10^5 ^50% Egg Infectious Dose (EID_50_) of the H1N1 influenza strain A/South Dakota/6/2007 which is 99% homologous to A/Brisbane/59/2007. This virus had been expanded in 10-day old chicken eggs at BIOQUAL (Rockville, MD) from a seed stock obtained from the CDC (Atlanta, GA). The diluted virus stock was inoculated intranasally (in a volume of 0.25 ml per nostril) into ferrets that had been anesthetized with ketamine/xylazine. Ferrets were observed for clinical signs twice daily while weight and temperature measurements were recorded once daily by technicians blind to treatment groups. Ferrets that lost more than 25% body weight or displayed severe clinical signs of infection (e.g. extreme lethargy or neurological impairment) were euthanized during the study. Remaining ferrets were euthanized on day 14 post challenge. Challenge experiments were performed in BSL-2 conditions and conducted at BIOQUAL, Inc. laboratories, which are approved for use by the USDA and CDC.

Nasal washes were obtained pre-challenge and on days 2,5, and 7 post-challenge, and viral titers were determined using a real-time PCR assay as previously described [34]. Briefly, RNA was isolated from nasal washes and RT-PCR was performed using TaqMan reagents (Applied Biosystems, Foster City, CA) along with primers and probes covering a highly conserved region within the nucleoprotein gene. The detection range for this assay is 20-10^7 ^copies/ml. Viral load data were analyzed using Tukey’s Honestly Significant Difference (HSD) method to determine differences between immunized groups and controls. For each analysis, P values < 0.05 were considered statistically significant.

## Results

### Particle size characterization

**Figure 1:**
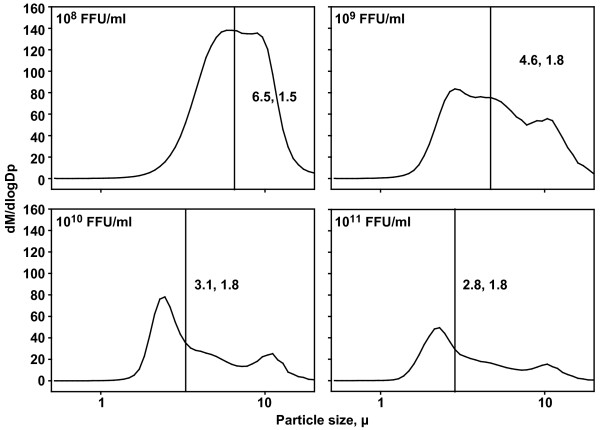
**Particle size analysis and dose optimization**. Graphs of particle size distributions of aerosolized Ad 11D LN05224 at logarithmic titers used (*FFU/ml*). Ordinate axis represents the mass of particles between *log*D*_p _*and *d log*D*_p _*(D*_p _*= particle diameter). The transecting line for each distribution represents the mass median aerodynamic diameter (MMAD) and geometric standard deviation (s_g_), respectively. The abscissa axis represents particle size in μm.

Characterization of the aerosolized Ad particle size was performed using a time-of-flight aerodynamic particle sizer (APS 3321, TSI Inc.). Proximity sampling was performed using the APS in a 16 liter plexiglass chamber. Resulting particle size distributions (Figure 1) showed MMADs fluctuated according to the relative concentration of the Ad present in the liquid carrier solution; the estimated MMAD ranged from 2.8 up to 6.5 μm, with relatively smaller MMAD ranges (3.1-2.8 μm) in generation events that incorporated higher Ad concentrations (10^10^-10^11^). The shifting MMAD is highlighted by a relatively stable geometric standard deviation (1.5-1.8) across all Ad concentrations used, indicating a minimal heterodispersity in the size distributions generated. Particle size limitations for respirability in the ferret is unknown, but the majority of mass represented in most of the size distributions shown would be considered respirable in species of similar size, weight, and anatomy [35].

### Viability of aerosolized adenoviral vectors

Results show the mean viral efficiency factor was relatively consistent over all concentrations, suggesting that the viability of the Ad will not be dramatically affected by the starting vector dose. Notably, the efficiency for this virus is not unusually low relative to the method of generation. A predictive dose delivered via inhalation can be derived from the mean viral efficiency factor, through estimation of the respiratory minute volume of the species, and the estimated aerosol concentration achieved with a particular viral starting concentration used. The nominal total titer administered to the ferrets was measured at 1.0E+10 PFU; the corresponding inhaled doses for the species by applying the viral efficiency factor estimate was 1.14E+04 PFU/animal.

### Antibody responses in ferrets immunized with Ad5 encoding HA

To compare the immune responses elicited by aerosol delivery to those by intramuscular injection, HI and pseudotype assays were performed on sera collected three weeks after the two immunizations by either route. As shown in Table 2, IM injection elicited moderately higher responses than aerosol delivery against both homologous and heterologous H1N1 viruses according to HI assays, although these differences were not found to be statistically significant via analysis by an unpaired t-test. Concurrently, the pseudotype inhibition assay also indicated that aerosol delivery elicited similar neutralizing responses as IM injection.

**Table 2:** Antibody titers of ferrets immunized with Ad5 encoding H1N1 HA.

	HI Assay (GMT)		Pseudotype Assay (IC50)	
	**NC**	**Brisbane**	**NC**	**Brisbane**

**Ad5-Control: AE**^**a**^	< 20	< 20	---	---

**Ad5-Brisbane (×2): AE**^**a**^	33	215	1064	2045

**Ad-Brisbane (×2): IM**^**a**^	98	390	1593	1805

^a ^N = 7

HI and pseudotype lentiviral assays were performed on ferret serum collected three weeks after the second immunization, against H1N1 strains of A/Brisbane/59/2007 and A/New Caledonia/20/99.

### Viral load reduction in ferrets challenged with H1N1 A/South Dakota/2007

**Figure 2:**
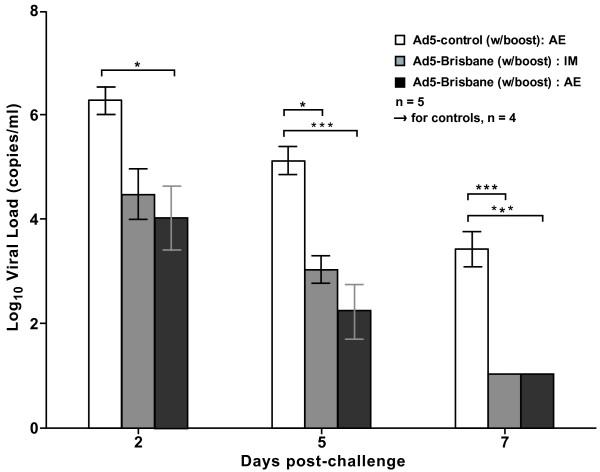
**Viral load reduction in H1N1 A/South Dakota/06/2007 challenged ferrets**. Viral loads were measured in H1N1-challenged ferrets at days 2,5 and 7 post-challenge. Bars indicate mean values of the log_10 _viral load, while error bars indicate standard error. Statistical differences were analyzed using Tukey's Honestly Significant Difference (HSD) method, and are indicated by asterisks where * represents a *p*-value between 0.05 and 0.01, ** indicates 0.01-0.001, and *** indicates < 0.001. Error bars indicate standard error.

To assess the extent of viral replication in the upper respiratory tract after challenge, nasal washes were performed at multiple time points following challenge and virus was quantified by RT-PCR (Figure 2). Significant reduction of viral loads was observed in aerosol-immunized groups compared to controls by day 2 post-challenge (pc) (*P *< 0.013). By day 5 pc, both immunization methods had significantly reduced viral loads compared to controls, (*P <*0 .001 for AE, *P = *0.007 for IM). The reduction of viral loads was more prominent on day 7 pc as both immunized groups reached the minimum detection level (20 copies/mL nasal wash) while the virus persisted in the control group.

## Discussion

Aerosol delivery is a potential alternative to parenteral injection for vaccine administration and may be advantageous in eliciting robust immune responses at the site of infection against respiratory pathogens such as influenza [7,8]. Here we characterize important factors for optimizing such delivery including particle size, vector viability, and actual delivered dose of an aerosolized Ad vector. We also compare the efficacy of aerosol vaccination to IM injection against a seasonal H1N1 challenge in ferrets.

Results from the particle size characterization experiment suggest that the concentration of the Ad solution has an effect on the size of the particle generated. This is important since particle size affects the site of deposition in the respiratory tract which will then influence biological responses [35]. The respiratory system and prevailing anatomy of the ferret is considered susceptible to particle sizes consistent with respirability estimates in mammals such as dogs and cats in contrast to similarly sized rodent species [36,37]. High concentrations of Ad resulted in generally smaller aerosolized particles despite the nebulizer's design to produce nominal 4 μm particles. This may be due to a clogging effect in which Ad precipitates on the nebulizing membrane, leaving residues which may hamper the efficiency of aerosolization. This hypothesis is supported by the observation that at high concentrations, the process of aerosolization took considerably longer than at low concentrations despite frequent changes and cleaning of nebulizing membranes. While other studies have similarly shown the effect of concentration on aerosol size [38], membrane "clogging" has not been previously documented and should be evaluated more closely.

Results from the seasonal influenza challenge study suggest that both aerosol and IM delivery of our Ad5 vaccine elicit similar levels of influenza-specific antibody responses, and are also comparable in reducing viral replication in the lungs of challenged ferrets. This is consistent with a previous study where we demonstrated robust immunogenicity and protection against lethal H5N1 influenza challenges in the ferret model [8] and is further supported by other recent challenge studies in mice [7,14]. While aerosolization may be comparable to IM delivery in serum antibody responses and viral load reduction, it may have significant advantages. One such advantage is the induction of local mucosal immune responses which may contribute to broader protection against heterologous strains and subtypes [4,7,8], whereas parenteral vaccination is incapable of inducing these types of responses [1]. In addition, aerosol delivery may enhance acceptability of vaccination since it does not require needles and causes less discomfort than intranasal sprays [7]. Lastly, aerosol delivery is a safe, reliable, and economically feasible vaccine delivery platform that does not have the attendant safety risks of injections.

## Competing interests

The authors declare that they have no competing interests.

## Authors' contributions

CJR participated in the design of the study and analysis of the data. AA participated in the coordination and conduct of the study. SKS conducted the study and analyzed the data. JPG conducted the study and drafted the manuscript. CJW, HA, and JG carried out the immunoassays. MR and SSR conceived of the study and analyzed the data. All authors read and approved the final manuscript.
